# Diversification and the rate of molecular evolution: no evidence of a link in mammals

**DOI:** 10.1186/1471-2148-11-286

**Published:** 2011-10-04

**Authors:** Xavier Goldie, Robert Lanfear, Lindell Bromham

**Affiliations:** 1 Centre for Macroevolution and Macroecology, Division of Evolution, Ecology and Genetics, Research School of Biology, The Australian National University, Canberra, A.C.T. 0200, Australia

## Abstract

**Background:**

Recent research has indicated a positive association between rates of molecular evolution and diversification in a number of taxa. However debate continues concerning the universality and cause of this relationship. Here, we present the first systematic investigation of this relationship within the mammals. We use phylogenetically independent sister-pair comparisons to test for a relationship between substitution rates and clade size at a number of taxonomic levels. Total, non-synonymous and synonymous substitution rates were estimated from mitochondrial and nuclear DNA sequences.

**Results:**

We found no evidence for an association between clade size and substitution rates in mammals, for either the nuclear or the mitochondrial sequences. We found significant associations between body size and substitution rates, as previously reported.

**Conclusions:**

Our results present a contrast to previous research, which has reported significant positive associations between substitution rates and diversification for birds, angiosperms and reptiles. There are three possible reasons for the differences between the observed results in mammals versus other clades. First, there may be no link between substitution rates and diversification in mammals. Second, this link may exist, but may be much weaker in mammals than in other clades. Third, the link between substitution rates and diversification may exist in mammals, but may be confounded by other variables.

## Background

Diversification is the net outcome of speciation and extinction. Clade size, the current species richness of a lineage, is a measure of net diversification because it is the result of the addition of species through speciation and the removal by extinction. A number of recent studies have shown positive relationships between rates of molecular evolution and net diversification. A positive relationship between substitution rates and species richness has been reported in angiosperms [1,2], carnivorous plants [3], and birds and reptiles [4,5]. Additionally, a relationship between the molecular path lengths of lineages and the number of nodes through which those lineages pass in molecular phylogenies has been interpreted as evidence of a connection between net diversification and rates of molecular evolution in a large range of taxa [6-8].

There are a number of possible causes of a relationship between rates of molecular evolution and net diversification. It has been suggested that elevated substitution rates in diverging populations are the result of changes to the selective and demographic landscape that accompany speciation [6,7]. Changed selective regimes at speciation could lead to elevated substitution rates at a number of loci as species adapt to new niches [9,10]. Strong reinforcing selection at hybrid contact zones, in particular, can lead to elevated substitution rates in genes associated with reproductive isolation [11-15]. Neutral loci linked to positively selected genes may also experience increased substitution rates at speciation events [16-18].

However, the majority of studies that report a link between net diversification and substitution rates focus on genes that are not obviously associated with traits under strong positive selection during speciation events. Rather, they tend to be based on "house-keeping" genes, such as metabolic genes (e.g. *CYTB, COIII, ND2, ALDOB*) and genes associated with transcription and translation (e.g. *16S rRNA, EEF2, MYC*) [4,5]. The observation that substitution rates at these loci are positively correlated to species richness suggests that genome-wide substitution rates are associated with net diversification.

It has been suggested that the process of speciation may cause increases in genome-wide substitution rates [7]. For instance, if small, fragmented and genetically isolated founder populations characterise most speciation events, slightly deleterious mutations may be fixed at an elevated rate due to reductions in the effective population size (*N_e_*) [19].

It is also possible the link between net diversification and rates of molecular evolution could be caused by differences in mutation rates between lineages. For instance, higher mutation rates, and subsequently elevated substitution rates, may lead to a more rapid acquisition of hybrid incompatibilities in diverging populations [20-22]. Given that hybrid incompatibilities accrue faster than linearly with the number of substitutions between diverging populations [23], even small differences in the underlying mutation rate could lead to relatively large differences in the number of incompatibilities between taxa, potentially resulting in more rapid reproductive isolation. In addition, elevated mutation rates may lead to higher levels of standing variation [24,25] available for divergent selection to act on during speciation, leading to the more rapid acquisition of local adaptations [11]. Elevated mutation rates could potentially influence net diversification by lowering extinction rates, for example by generating standing variation on which selection for adaptation to environmental change can act [26].

Finally, there may be no direct causal link between rates of molecular evolution and net diversification. Instead, the association between may be caused indirectly by co-variation between molecular evolutionary rates, diversification and other traits and processes. Shorter generation time, higher fecundity and shorter life-spans have all been linked to substitution rates in mammals [24,27-29]. If these processes independently influence the process of diversification, this may lead to a non-causal association between substitution rates and net diversification. For instance, it has been suggested that larger bodied mammals have a higher extinction risk due to the effect of reduced reproductive rates and low population densities [30,31]. Consequently, if extinction rates determine clade size, larger bodied animals may characterise smaller clades. This could lead to an indirect positive association between clade size and substitution rates.

Methodological artifacts could also cause an association between rates of molecular evolution and diversification. For example, it has been suggested that the node density effect, where molecular branch-lengths which pass through more nodes tend to be longer, could be responsible for the association between rates of molecular evolution and diversification in some studies [32]. However an association between rates and diversification has also been noted in studies that controlled for the node density effect [5].

Mammals provide an ideal opportunity to investigate the generality and potential direction of causality of the relationship between net diversification and rates of molecular evolution. A considerable amount of research has been conducted investigating the relationship between substitution rate variation and life history in mammals [27,28,33,34]. In particular, body size, generation time and longevity have been shown to be associated with substitution rates [27,28,34]. The availability of a large amount of life history data for mammals permits their inclusion in this study as a potentially confounding factor [35]. Additionally, phylogenetic relationships are well studied in mammals [36-42], allowing independent sister-clades to be chosen with some confidence.

In this study, we use phylogenetically independent comparisons of sister clades to test for an association between substitution rate and clade size in mammals. Using protein-coding genes from both nuclear and mitochondrial genomes, we test for a relationship between clade size and total substitution rates (*T*), synonymous substitution rates (*dS*), non-synonymous substitution rates (*dN*), and the ratio of *dN *to *dS *(*ω*).

These measures provide a way in which to examine the different processes that may cause rates of molecular evolution to co-vary with clade size. Synonymous mutations do not change the encoded amino acid sequences, and while not necessarily neutral [43,44], are expected to have sufficiently small selection co-efficients [25,43], for differences in *dS *between species to closely reflect underlying differences in mutation rates [45]. Non-synonymous mutations, by contrast, change the encoded amino acid sequences. These changes are more likely to be affected by the interaction between selection and effective population size (*N_e_*), that is, slightly deleterious non-synonymous substitutions are expected to be fixed in populations of smaller *N_e _*at a greater rate than in larger populations [45]. As a result, *dN *is expected to be influenced by *N_e_*, selection and mutation rates. Consequently, higher values of *ω *may reflect reduced *N_e _*or increased positive selection.

If positive selection or reductions in *N_e _*at speciation events were responsible for the link between net diversification and substitution rates, then we would expect to observe a positive relationship between *ω *and clade size. This is because both positive selection and reductions in *N_e _*should increase the fixation rate of non-synonymous mutations, but are unlikely to greatly influence the rate of fixation of synonymous mutations. By contrast, if higher net diversification was an outcome of elevated mutation rates causing more rapid reproductive isolation and divergence, then we would expect to observe positive relationships between all measures of substitution rate and clade size, but not necessarily a relationship between *ω *and clade size [5].

## Methods

### Sister-Pairs

We used phylogenetically independent [46] sister-pairs of clades to investigate the relationship between substitution rates and clade size, using both nuclear and mitochondrial sequences. Each of the two clades in a sister-pair has had, by definition, the same amount of time since their most recent common ancestor to accumulate both species and genetic change. Thus, any difference in species numbers between the sister-pair reflects a difference in net diversification since their last common ancestor. Similarly, difference in the average substitution rate since their most recent common ancestor should be reflected as a difference in molecular branch length between a sister-pair [1]. Each sister-pair is independent of other such pairs, and therefore fulfills the requirement of independence for subsequent statistical analyses [46,47].

We used published phylogenies to select our phylogenetically independent sister-pairs and their nearest available out-groups. We excluded any potential sister-pairs for which a reciprocally monophyletic relationship between the two clades was not well supported in the literature. References in support of each sister-pair in our analyses are included in Additional File 1.

### Mitochondrial Sister-Pairs and Sequence Data

For our mitochondrial analyses we investigated the relationship between clade size and substitution rates using 28 sister-pairs of clades, corresponding approximately to family level contrasts. Our mitochondrial dataset also provided the additional opportunity to perform analyses on deeper (n = 9 pairs) and shallower (n = 27) sister-pairs of clades, to test whether the relationship between clade size and substitution rate differed with the taxonomic level of the clades [48]. Details of these sister-pairs are included in Additional File 1.

For mitochondrial analyses, we used all protein coding genes from the heavy strand of whole mitochondrial genomes available from GenBank (*ND1, ND2, ND3, ND4, ND4L, ND5, COI, COII, COIII, ATP6, ATP8 *and *CYTB*). We removed regions of coding overlap shared by mitochondrial genes (*ATP8*-*ATP6, ATP6*-*COIII, ND4L-ND4*).

**Figure 1:**
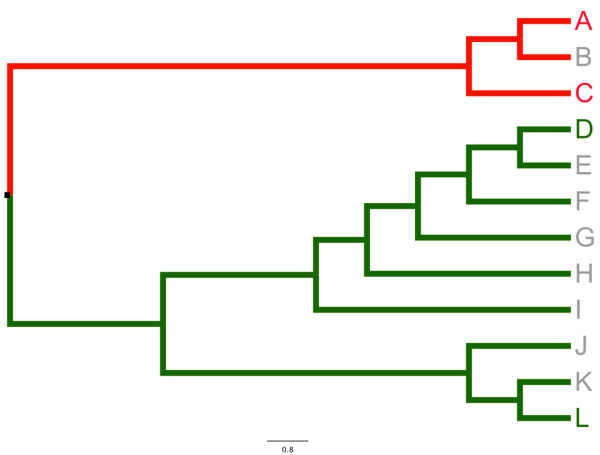
**Sequence selection methods**. A sister-pair comprising more speciose (green) and less speciose (red) clades. Coloured taxa indicate those for which sequence data is available. Using our methods, Taxon D is selected for analysis, because its root-to-tip branch is separated from the basal node by 6 nodes, compared to 3 for Taxon L. By contrast Taxon A is selected for analysis using our methods because its root-to-tip branch is separated from the basal node by two internal nodes, compared to one for Taxon C. In both cases, a large component of the sequences are shared by other members of the respective clades over the whole molecular branch length, relative to the sister clade.

To avoid the node density effect in maximum likelihood substitution rate estimates [32,49], we used a single mitochondrial genome sequence to represent each clade. A single sequence can be used to estimate representative substitution rates for a clade because a number of the substitutions from that sequence will occur on internal (shared) branches (Figure 1). Although some potential data are excluded using this method, it reduces the likelihood that substitution rate estimates are biased by the node density effect [5].

Where more than one mitochondrial genome sequence was available on GenBank for a given clade, we selected the sequences based on the number of internal nodes in the published molecular phylogenies used to select the sister clades. In the more speciose clade, we chose the sequence with the greatest number of internal nodes. In the less speciose clade, we selected the sequence with the fewest number of internal nodes (shown in Figure 1). We did this in order to maximize the potential difference in number of cladogenetic events, and thus to increase the power to detect any difference in branch length due to lineages undergoing cladogenesis [6,8], without reconstructing those nodes in the estimation of rates, which may lead to node density effect [32].

### Nuclear Sister-Pairs and Sequence Data

For our nuclear data, we investigated the relationship between substitution rate and clade size using 31 sister-pairs of clades, corresponding to approximately family-level contrasts. We also tested for relationships between clade size and substitution rate within specific groups of mammals, as it has been shown that patterns of substitution rate variation and patterns of diversification can differ between these groups [27,28]. Consequently, we tested for a relationship between clade size and substitution rate independently for the Eutheria (n = 22 pairs) and the Metatheria (n = 7). Details of these sister-pairs are included in Additional File 1.

For our nuclear analyses, we used nuclear genes obtained from GenBank. There was a substantial trade-off between taxonomic and genetic coverage for nuclear gene sequences. In order to optimise both of these (and thus optimise power in subsequent regression analyses), different sets of nuclear genes were chosen for different groups. Our whole mammalian analysis (n = 31) included *BRCA1, RAG1 *and *VWF *(2850 bp); our eutherian analysis (n = 22) included *ADORA3, ATP7A, BDNF, BRCA1, RAG1, RAG2 *and *VWF *(4302 bp); and our metatherian analysis (n = 7) included *APOB, BRCA1, IRBP, RAG1 *and *VWF *(4255 bp). These genes were the most widely sampled nuclear protein coding sequences available on GenBank. Accession numbers for nuclear gene sequences are contained in Additional File 1.

As with our mitochondrial analysis, to reduce the impact of the node density effect in maximum likelihood substitution rate estimates we used a single representative nuclear gene sequence for each clade. We used the same selection criteria for selecting our sequences where more than one sequence was available for a gene within a given clade. In some instances, we were unable to obtain all nuclear gene sequences from a single species to represent a given clade. In these instances, we constructed chimeric sequences, where gene sequences were sourced from different species within a single clade. In doing so, we selected species that were as closely related as possible.

### Substitution Rate Estimates

We used *HyPhy v1.0b *[50] to estimate total (*T*), synonymous (*dS*) and non-synonymous (*dN*) branch lengths on the sister pairs shown in Additional File 2. We used the Muse and Gaut [51] model of codon substitution (MG94), coupled with a general time reversible model of sequence evolution, with codon frequencies estimated from the data in a 3 × 4 matrix (that is, frequencies of bases were estimated for each codon position). This model is denoted the *MG94xREV_3 × 4_DualRV *model in *HyPhy *notation. The dual rate variation models in *HyPhy *explicitly accounts for variation in *dS *across both lineages and sites, potentially allowing for more accurate estimates of both *dS *and *ω *than other methods [52,53]. We used the Akaike Information Criterion (AIC) to determine whether our datasets should be partitioned [54]. For the nuclear sequences, the best AIC score was obtained with separate *MG94xREV_3 × 4_DualRV *codon substitution models, equilibrium frequencies, and rate parameters estimated for each gene. For the mitochondrial sequences, the best AIC score was obtained with a single *MG94xREV_3 × 4_DualRV *codon substitution model estimated for all genes combined. Estimates of *T, dN *and dS, were calculated for each branch of the phylogeny; the latter two were used to calculate *ω*. However, only the substitution rate and *ω *estimates for terminal branches were retained for use in subsequent analyses [55].

### Clade Size

We used extant clade size as a measure of net diversification for our analyses. Previous research investigating these relationships have used varied metrics to represent diversification, including extant clade size [1,5], node number [6,8] and diversification rate [4]. Differences in extant clade size between sister clades - which are by definition the same age - are measures of differences in the net diversification rates of those clades. We calculated extant species numbers for each clade in each sister-pair from Wilson and Reeder's *Mammal Species of the World *[56], ensuring also that species numbers reflected any changes to taxonomy within more recent systematics literature. Species numbers for each clade are given in Additional File 1.

### Body size

Substitution rates in mammals are known to be influenced by a number of life history variables, including generation time [29], fecundity [27], and longevity [57]. These life history variables, which are correlated with body size [58,59], have also been suggested as candidate variables influencing net diversification in mammals [60-63]. It is possible that an association between substitution rates and clade size may be the result of both net diversification and substitution rates co-varying independently with these life-history variables. We tested for these indirect associations between clade size and substitution rate by including body size in our analyses.

We calculated body mass contrasts for each sister pair used in our analyses. We obtained body mass values for most species in each clade from the panTHERIA database [35]. For eight species for which a value was not available in panTHERIA, we sourced body mass estimates from the literature. Where more than one estimate for a species was available in the primary literature, we took the arithmetic mean for all available estimates for the species, weighted by the sample sizes of the estimates, and excluding extreme minimal and maximal values. These data, together with references, are available in Additional File 3.

We used the maximum likelihood estimator (MLE) of Welch and Waxman [55] to calculate body mass contrasts for each sister pair. The MLE uses suitably transformed (in this case, log transformed [64]) body size values with the phylogeny of the sub-tree defined by the most recent common ancestor of the two clades to calculate time-averaged differences in body mass between clades. We used a number of source phylogenies for these estimates [39,41,61,65,66]. This maximum likelihood estimation method has advantages over simple averages of body sizes (tip measurements) across a clade, in that it is less prone to the effect of extreme values; provides robust estimates where data may be missing (i.e. unmeasured tips); and takes into account the evolution of the trait over a clade's evolutionary history.

### Statistical Tests

#### Testing for Substitution Rate Variation

We tested whether our alignments contained significant variation in substitution rates between terminal lineages. We compared the likelihoods of two models: an equal-rate model, where terminal branches within a pair are constrained to have equal substitution rates, but substitution rates are allowed to vary between pairs; and a free-rate model, where a separate substitution rate is estimated for each terminal branch. We calculated the likelihood of each of these models using the phylogenies shown in Additional File 2. We used Akaike information criterion scores (AIC) to compare the likelihoods of the two models [54]. We took a difference in AIC (ΔAIC) scores of 10 units as our threshold for significance, where ΔAIC < 10 failed to reject the null hypothesis of no difference in substitution rates. Details of this analysis are included in Additional File 4.

#### Linear regressions

We tested for associations between differences in clade size, body size and substitution rates, using linear regressions forced through the origin [47,67]. Differences in the variables for each sister pair were calculated as ln(V_*A*_)-ln(V_*B*_), where ln(V_*i*_) represents the log-transformed variable for Clade *i*. Log transformation of the variables was necessary to meet the assumptions of parametric regressions. Diagnostic tests recommended by Freckleton [68] indicated that these transformations were appropriate.

More distantly diverged sister-pairs are associated with more evolutionary change, and thus tend to generate contrasts of larger magnitude; this can lead to unequal variance between data points [47,67], which violates the assumptions of parametric statistical tests. To account for this, we standardised differences in all variables by weighting each contrast by a measure of the pair's genetic divergence. We determined that the square root of the sum of the pair's total substitution branch length values was suitable as a measure of standardisation: (*T*_A _+ *T*_B_)^0.5^. We used the diagnostic methods recommended by Garland [67] to confirm that these standardisations were appropriate for the data to meet assumptions of linear regression. Contrasts were excluded from the analysis where diagnostic tests indicated that the differences in substitution rates could not be reliably estimated from the molecular branch lengths, either because the contrasts were too shallow, or their substitution rates too slow [27,55], or their substitution rates saturated (i.e. > 1 substitutions per site for *T*; > 1 substitutions per codon for *dN *and *dS*). Details of which data points were removed for each analysis are indicated in Additional File 1.

To verify that our results were not dependent on the transformations or standardisations used, all statistics were also performed on non-transformed and non-standardised data, and the results did not differ. All statistics and diagnostic tests were performed in R [69].

#### Correction for multiple tests

Our analysis resulted in a number of tests of three hypotheses: *dN, dS, T *and *ω *are associated with clade size; *dN, dS, T *and *ω *are associated with body size; and clade size is associated with body size. Weighted Z tests were used to address the issue of multiple testing [70]. A weighted Z test combines tests of the same hypothesis to assess the support for that hypothesis across different datasets. To combine tests of the same hypothesis performed on different datasets, the P values from the individual regressions are first converted to one-tailed P values. In this instance, we converted P values from regressions (two-tailed) to one-tailed values by assuming that substitution rate would be positively associated with clade size (as observed by [1,4-6,70]) and negatively associated with body mass (as observed by [27]). Values were then converted to individual Z-scores. We then calculated an overall weighted Z-score, weighting each individual Z-score by the degrees of freedom in each test,[70]. Weighted Z-scores were then used to calculate overall P values for the combined test for each hypothesis.

In combining our tests of hypotheses of clade size against measures of rates of molecular evolution, *T, dN, dS *and *ω *were treated separately, given that we were testing for the effect of each independently on clade size in our analyses. For example, we combined the P values for tests of *dN *against clade size, from both nuclear and mitochondrial datasets. For tests of body size against measures of rates of molecular evolution, *dN, dS *and *T *and were also treated separately. Details of the Z-tests are included in Additional File 5.

## Results

### Evidence of Substitution Rate Variation

A free-rate model, where a separate substitution rate was estimated for each branch, had significantly better fit to the data for 4 of our 6 alignments, over an equal-rate mode where terminal branches within a pair had equal substitution rates. Free-rate models for *dN, dS *and *T *all had a significantly better fit to the data for these alignments; only results for *T *are shown. For two of our alignments (mitochondrial shallow, nuclear metatherian), an equal-rate model had significantly better fit to the data over a free-rate model. Equal-rate models for *dN, dS *and *T *were all significantly preferred for these alignments; only results for *T *are shown. Details of this analysis are included in Additional File 4.

### Mitochondrial Data

There were no significant associations between *T *or *dN *or *dS *and clade size for our 28 approximately family level mitochondrial contrasts of mammals (Table 1), nor for deeper (n = 9, Table 2) or shallower (n = 27, Table 3) contrasts. Mitochondrial *dS *estimates were saturated for the majority of taxa (Family: 27/28; Deep: 9/9; Shallow: 24/27), making tests of their association with clade size and body size unreliable. We attempted to address this issue by measuring rates of synonymous transversion at RY coded four-fold degenerate sites. However, we were not able to detect the expected relationship between synonymous transversion rates and body size [27]. As such we did not consider that these measures of substitution rate had sufficient power, and we do not address them further.

**Table 1:** Mitochondrial Family (Approximately) Level Contrasts

Response Variable	Predictor Variable	Coefficient	R^2^	**d.f**.	P value
ln(Clade Size)	ln(*dN*)	-1.1185	0.1279	27	0.066
ln(Clade Size)	ln(*T*)	-0.6133	0.0128	27	0.560
ln(Clade Size)	ln(*dS*)^#^	-0.0865	0.0867	16	0.236
ln(Clade Size)	ln(*ω) *^#^	-0.0077	0.2127	16	0.054
ln(Clade Size)	ln(Body Size)	0.1398	0.0130	27	0.545
ln(*dN*)	ln(Body Size)	-0.0073	0.0003	27	0.921
ln(*T*)	ln(Body Size)	0.0017	0.0001	27	0.968
ln(*dS*)^#^	ln(Body Size)	-0.2046	0.0024	16	0.846
ln(*ω*)^#^	ln(Body Size)	-0.2721	0.1371	16	0.130

**Table 2:** Mitochondrial Deep Level Contrasts

Response Variable	Predictor Variable	Coefficient	R^2^	**d.f**.	P value
ln(Clade Size)	ln(*dN*)	-0.6282	0.0199	8	0.698
ln(Clade Size)	ln(*T*)	2.8130	0.1062	8	0.358
ln(Clade Size)	ln(Body Size)	-0.4004	0.0666	8	0.472
ln(*dN*)	ln(Body Size)	-0.0117	0.0011	8	0.927
ln(*T*)	ln(Body Size)	-0.0339	0.0351	8	0.602

**Table 3:** Mitochondrial Shallow Level Contrasts

Response Variable	Predictor Variable	Coefficient	R^2^	**d.f**.	P value
ln(Clade Size)	ln(*dN*)	0.2991	0.0051	25	0.722
ln(Clade Size)	ln(*T*)	-0.5455	0.0069	25	0.683
ln(Clade Size)	ln(*dS*)^#^	-1.0500	0.1087	23	0.107
ln(Clade Size)	ln(*ω) *^#^	-0.0035	0.0010	23	0.88
ln(Clade Size)	ln(Body Size)	0.1566	0.0278	24	0.416
ln(*dN*)	ln(Body Size)	0.0006	6 × 10^-6^	24	0.990
ln(*T*)	ln(Body Size)	-0.0444	0.0960	24	0.123
ln(*dS*)^#^	ln(Body Size)	0.0280	0.0074	23	0.683
ln(*ω*)^#^	ln(Body Size)	-1.1831	0.0159	23	0.553

Tables 1, 2 and 3 - Regressions between rates, clade size and body size for mitochondrial sequence data

Traits are measured as differences in values between sister-pairs of mammalian clades. Co-efficient: estimated co-efficient of the predictor variable; R^2 ^= co-efficient of determination; d.f: degrees of freedom in model. Synonymous substitution rates and dN/dS ratios (*ω*) estimated in *PAML *indicated with ^#^; all other rates were estimated in *HyPhy*. P value: significance of value of model; Significance: * = P < 0.05, ** = P < 0.005.

In case synonymous substitution rates were overestimated by the particular model in *HyPhy*, we re-estimated our mitochondrial rates in *PAML v4.4 *[71] using a codon-based substitution model of Goldman and Yang [72]. Both the synonymous and non-synonymous codon substitution rates were allowed to take branch-specific values. We subsequently obtained fewer saturated synonymous substitution rates for the approximately family level (11/28) and shallower contrasts (3/28); all of our deeper contrasts remained saturated. We did not find a significant relationship between clade size and *dS*, or *ω *for these re-estimated data (Tables 1, 2 and 3). However, we also did not detect the expected positive relationship between *dS *and body size [27], indicating our data most likely did not have sufficient power.

Therefore, as a *post hoc *analysis, we used mitochondrial *dS, dN, and ω *estimates for mammalian sister clades from Welch *et al*. [27] to test for a clade size effect, in order to maximise our power to detect these relationships. This dataset contains mammalian sister pairs from varying taxonomic depths (1.4 MYA - 74.1 MYA), covering ~9,500 bp of mitochondrial protein coding sequences. Branch specific codon substitution rates were estimated by the authors in *PAML *[71]. From that dataset, we excluded pairs that did not have support in the literature as reciprocally monophyletic sister clades to the exclusion of all the other pairs, or where we were unable to determine clade sizes. We also excluded pairs excluded by the original authors due to their failure to meet the assumptions required for linear regressions. We then calculated species numbers for each member of each sister-pair and standardised them according to Welch *et al.*'s [27] methods (Details in Additional File 1). We calculated MLE body mass contrasts for each sister pair of clades. We excluded *dS *estimates that were saturated. There were no significant associations between clade size and substitution rate (*dN *or *dS*), or between clade size and *ω *in these analyses (Table 4).

**Table 4:** Welch *et al*. [27] Mitochondrial Contrasts

Response Variable	Predictor Variable	Coefficient	R^2^	**d.f**.	P value
ln(Clade Size)	ln(*dN*)	-0.2485	0.0064	42	0.605
ln(Clade Size)	ln(*dS*)	-1.4968	0.1031	26	0.096
ln(Clade Size)	ln(*ω*)	0.4371	0.0179	27	0.423
ln(Clade Size)	ln(Body Size)	0.0783	0.0066	42	0.600
ln(*dN*)	ln(Body Size)	0.0545	0.0306	42	0.256
ln(*dS*)	ln(Body Size)	-0.1263	0.1728	25	0.031 *
ln(*ω*)	ln(Body Size)	0.0586	0.0338	36	0.269

**Table 4 - Regressions between rates, clade size and body size for mitochondrial sequence data of Welch *et al***. [27]

Traits are measured as differences in values between sister-pairs of mammalian clades. Co-efficient: estimated co-efficient of the predictor variable; R^2 ^= co-efficient of determination; d.f: degrees of freedom in model; P value: significance of value of model; Significance: * = P < 0.05, ** = P < 0.005.

We did not detect a significant relationship between body size and our estimates of *T *or *dN *substitution rates calculated in *HyPhy *(Tables 1, 2 and 3). Mitochondrial *dS *rates have previously been shown to be negatively associated with body size [27,29]. We did not detect this relationship between body size and *dS *and *ω*, using our non-saturated *dS *rates re-estimated in *PAML*. However, we could detect the previously reported relationship between body size and *dS *estimates from the data of Welch *et al.*, [27] (Table 4).

There were no significant associations between body size and clade size in any of our mitochondrial datasets.

### Nuclear Data

We did not find any association between clade size and any of the measures of substitution rate (*T, dN*, dS) estimated from our nuclear gene data set for 32 mammalian sister pairs (Table 5). We found a significant positive association between total substitution rate (*T*) and clade size in the Eutheria-only data set (R^2 ^= 0.1857, P = 0.0453: Table 6). However, this relationship was not detected in analyses of clade size against *dN *or *dS *for the Eutheria-only data, and is not significant when corrected for multiple tests (see below). Our Metatheria-only analysis did not produce any significant association between substitution rate and clade size (Table 7). We did not find any association between *ω *and clade size in any of our nuclear datasets.

**Table 5:** Mammalia Nuclear Contrasts

Response Variable	Predictor Variable	Coefficient	R^2^	**d.f**.	P value
ln(Clade Size)	ln(*dN*)	-0.5432	0.0252	25	0.421
ln(Clade Size)	ln(*dS*)	0.0987	0.0034	26	0.765
ln(Clade Size)	ln(*ω*)	-1.2561	0.1022	26	0.097
ln(Clade Size)	ln(*T*)	-0.4149	0.0094	23	0.645
ln(Clade Size)	ln(Body Size)	0.0414	0.0022	31	0.793
ln(*dN*)	ln(Body Size)	-0.1062	0.1569	25	0.041 *
ln(*dS*)	ln(Body Size)	-0.1292	0.2794	25	0.004 **
ln(*ω*)	ln(Body Size)	0.0287	0.0154	26	0.529
ln(*T*)	ln(Body Size)	-0.1237	0.3384	22	0.002 **

**Table 6:** Eutheria Nuclear Contrasts

Response Variable	Predictor Variable	Coefficient	R^2^	**d.f**.	P value
ln(Clade Size)	ln(*dN*)	0.8154	0.05681	18	0.312
ln(Clade Size)	ln(*dS*)	1.2337	0.1111	18	0.151
ln(Clade Size)	ln(*ω*)	-1.6541	0.1499	15	0.125
ln(Clade Size)	ln(*T*)	1.8792	0.1839	20	0.0453 *
ln(Clade Size)	ln(Body Size)	-0.1453	0.0308	22	0.4123
ln(*dN*)	ln(Body Size)	-0.2412	0.3446	18	0.0065 *
ln(*dS*)	ln(Body Size)	-0.1759	0.2149	18	0.0395 *
ln(*ω*)	ln(Body Size)	0.1272	0.1433	15	0.134
ln(*T*)	ln(Body Size)	-0.0925	0.2397	20	0.0208 *

**Table 7:** Metatheria Nuclear Contrasts

Response Variable	Predictor Variable	Coefficient	R^2^	**d.f**.	P value
ln(Clade Size)	ln(*dN*)	2.2613	0.1736	6	0.304
ln(Clade Size)	ln(*dS*)	-1.6842	0.2381	6	0.221
ln(Clade Size)	ln(*ω*)	1.5536	0.3391	6	0.132
ln(Clade Size)	ln(*T*)	-2.6421	0.1202	6	0.401
ln(Clade Size)	ln(Body Size)	0.7831	0.3792	6	0.14
ln(*dN*)	ln(Body Size)	0.1231	0.2758	6	0.181
ln(*dS*)	ln(Body Size)	-0.0644	0.0306	6	0.679
ln(*ω*)	ln(Body Size)	0.1876	0.1549	6	0.335
ln(*T*)	ln(Body Size)	-0.0094	0.0031	6	0.895

Tables 5, 6 and 7 - Regressions between rates, clade size and body size for nuclear sequence data.

Traits are measured as differences in values between sister-pairs of mammalian clades. Co-efficient: estimated co-efficient of the predictor variable; R^2 ^= co-efficient of determination; d.f: degrees of freedom in model; P value: significance of value of model; Significance: * = P < 0.05, ** = P < 0.005.

Body size was significantly negatively associated with *T, dN *and *dS *for the whole mammalian and Eutheria-only nuclear data sets (Tables 5 and 6), but not for the Metatheria-only data. There were no relationships between *ω *and body size in any of the nuclear data sets. Body size was not significantly associated with clade size in any of the nuclear data sets.

The MLE method of body mass contrast estimation assumes homogeneity of variance in body size between both clades in a sister pair. We found that this assumption was not valid for a minority of contrasts (Additional File 1). Consequently, we also calculated body mass contrasts based on the logarithm of geometric means of sister clades - an approach which does assume that sister clades have homogeneous variance in body size. We tested whether the MLE contrasts and geometric mean contrasts for each sister pair were significantly different using a paired t-test. None of the datasets had significant differences between the MLE contrasts or geometric mean contrasts (Additional File 1). Furthermore, results of all regressions were qualitatively identical using contrasts calculated with either approach.

### Correction for multiple tests

Weighted Z tests indicate there is no association between clade size and *ω, T, dN *or *dS *across all data-sets (Table 8), identifying the association between Eutheria-only total substitution rate (*T*) and clade size as a likely false positive. By contrast, weighted Z tests indicate that there is a negative association between body mass and substitution rate estimates, except for the pooled (i.e. mitochondrial and nuclear) *dN *data (*dN*: P = 0.2297, *dS*: P = 5.20 × 10^-5^, *T*: P = 7.3 × 10^-4^; Table 4). However, previous studies have indicated that mitochondrial *dN *rates are not associated with body mass [27]. When these *dN *results are separated into mitochondrial and nuclear data sets, weighted Z tests show a significant negative association between body mass and nuclear *dN *(P = 0.0017; Table 4), but not mitochondrial (P = 0.7873), consistent with these previous results. There was no significant relationship between *ω *and body mass across all datasets (P = 0.1026; Table 4).

**Table 8:** Z Test Results on Multiple P Values

Response	Predictor	n	Weighted Z	P value
Clade Size	*T*	6	-0.1190	0.4526
Clade Size	*dN*	7	0.9506	0.8291
Clade Size	*dS*	6	1.4619	0.9281
Clade Size	*ω*	6	0.8970	0.8151
Clade Size	Body Size	7	0.7700	0.7794
*dN*	Body Size			
*All*		7	-0.7400	0.229
*Nuclear*		3	-2.9344	0.0017 **
*Mitochondrial*		4	0.7970	0.7873
*dS*	Body Size	6	-3.2518	0.000573 **
*T*	Body Size	6	-3.2421	0.000593 **
*ω*	Body Size	6	-1.2667	0.1026

Table 8 - Results of weighted Z tests for multiple comparisons.

Weighted Z: the combined weighted value for multiple Z scores for each individual test; n: number of tests across which Weighted Z score was calculated; P value: significance of Weighted Z score. Significance: * = P < 0.05, ** = P < 0.005.

## Discussion

We have found no evidence for a link between net diversification and substitution rate in mammals. We did not find a significant relationship between clade size and total substitution rate (*T*), non-synonymous substitution rates (*dN*), or synonymous substitution rates (*dS*) for any of our mitochondrial or nuclear datasets. These results are in contrast to results of similar studies on other taxa, which have shown a positive relationship between rates of molecular evolution and clade size in angiosperms [1], birds [4,5], and reptiles [4], and a positive relationship between molecular branch lengths and the number of nodes through which those branches pass in a large range of taxa [6,8].

There are a number of explanations for our failure to detect a relationship between substitution rates and clade size in mammals: (1) the relationship exists but our analyses do not have the power to detect it; (2) the relationship exists, but is confounded by other processes in mammals; and (3) the relationship between clade size and substitution rates is not universal and does not exist in mammals.

We cannot rule out a lack of power producing the results we report here, but we do not consider this the most likely explanation for our results. We were able to detect a significant relationship between body size and substitution rates in both our nuclear data and the mitochondrial data from Welch *et al *[27], indicating that the data used here have the power to detect associations between substitution rate and life history variables. Given the previously reported strength of the association between clade size and substitution rates in other groups (angiosperms, 89 comparisons, ~5 kbp [1]; reptiles, 16 comparisons ~10 kbp DNA [4]; and birds, 12 comparisons and ~10 kbp for mtDNA [4], 32 comparisons and ~17 kbp for nuclear DNA [5]), the lack of a significant relationship between substitution rate and clade size in our data (42 comparisons and ~10 kbp for mtDNA, 31 comparisons and ~3 kbp for nuclear DNA) suggests that this relationship is either weak or absent in mammals.

It is possible that there is an association between substitution rates and clade size in mammals, but that this relationship is masked by interactions with other variables. For instance, it has been suggested that abundance (measured as group size or population density) is positively linked to diversification rate in mammals [62]. If abundance is also correlated to effective population size, then more abundant mammal species could have reduced rates of non-synonymous substitution, since slightly deleterious mutations have lower fixation probabilities in larger populations [45,73]. So it is possible that more abundant mammal species have both higher net diversification and lower substitution rate, and that these relationships could confound our ability to observe a positive link between net diversification and the substitution rate. However, if the link between diversification and molecular evolution is confounded by effective population size, we might expect to detect an association between *ω *and clade size, which we have not seen in this study.

Perhaps a more likely explanation for the lack of an association between substitution rates and clade size in mammals is that the relationship does not exist for this group. Previous explanations of the association between rates of molecular evolution and clade size have focused on three possible causes: (i) speciation causes increases in substitution rates; (ii) mutation rates drive diversification; and (iii) both diversification and substitution rate are linked to another factor.

Some previous studies have explained a positive association between net diversification and substitution rate as the result of the demographic and selective processes characterising speciation [7]. Specifically, more frequent speciation events could be expected to lead to reductions of the long term *N_e _*in more rapidly speciating clades [6,8]. Reductions in long term *N_e _*would be expected to increase the fixation rate of nearly neutral mutations (i.e. those with selection co-efficients approaching 1/*N_e_*) [73], and thus increase the non-synonymous substitution rate. If this is the cause of the previously noted link between diversification and rates of molecular evolution then it is possible that the connection between speciation events and substitution rate is for some reason not as strong in mammals. For example, it is possible that frequent population size fluctuations in mammals overwhelm any signal of population size reduction associated with speciation events.

A recent study indicated that the correlation between substitution rate and clade size in birds might be driven by the effect of mutation rates on the process of diversification [5]. Hybrid fitness in birds has been shown to be inversely proportional to genetic distances between parents [74-77], possibly supporting a significant role for the accumulation of Dobzhansky-Muller incompatibilities in speciation in birds [20,21]. If this is the case, then the rate of formation of species through post-zygotic hybrid incompatibility might be influenced by the mutation rate [22,23]. It has been suggested that hybrid incompatibilities in mammals develop at a much faster rate than in birds [78], possibly due to higher rates of regulatory evolution [78,79]. If reproductive isolation in mammals is determined to a greater degree by adaptive divergence at regulatory and developmental loci (such as those loci associated with placentation, genomic imprinting or mediating viviparity driven conflicts [80-82]), then the molecular change accompanying speciation may be predominantly in a few key loci, rather than due to the accumulation of genome-wide incompatibilities.

It is also possible that the positive association between rates of molecular evolution and clade size observed in some taxa is not due to a direct effect of speciation on molecular evolution, or vice versa, but the result of another variable driving both processes independently of each other, leading to an indirect correlation between the two.

Many life-history correlates of substitution rate in mammals have been identified [27,29,57], however, few of these life history traits have been shown to consistently scale with mammalian clade size. The life-history traits that scale with substitution rates in mammals (generation time, fecundity, and longevity) also correlate tightly with body size [57-59]. Because of this, body size is significantly negatively associated with substitution rates, as demonstrated both here and in other studies [24,27,29,53,59]. If extinction rates increase with body size, it could reduce the clade size of larger-bodied taxa potentially leading to an indirect positive relationship between substitution rates and clade size. However, a consistent relationship between body size and clade size in mammals has not been established - we find no evidence for such a relationship in this study, and the results of other studies are equivocal and inconsistent across different clades of mammals [60,62,63,83]. Taken together these results suggest that it is unlikely in mammals that body size, or life history traits that correlate with size, drives both substitution rates and diversification (via extinction or speciation) rates, as may be the case in other taxa [5,83].

## Conclusions

Contrary to patterns observed in other taxa, we have not detected a relationship between clade size in mammals and substitution rate, measured from total, synonymous and non-synonymous substitution rates in both nuclear or mitochondrial genes. Given that our study is likely to have comparable power to other similar studies, these results suggest that any association between net diversification and substitution rate is either absent or very weak in mammals.

## Authors' contributions

XG, RL and LB designed the analyses; XG performed the analyses; XG, RL and LB wrote the manuscript. All authors have read and approved the final manuscript.

## Supplementary Material

Additional file 1 **Nuclear and Mitochondrial Data**. Excel spreadsheet containing substitution rate estimates, estimates of body size differences between sister-pairs, estimates of species number (clade size), Accession Numbers and references. Click here for file

Additional file 2 **Phylogenies**. PDF document containing phylogenies used for all analyses described in the main text. Click here for file

Additional file 3 **Body Mass Data**. PDF document containing body mass data and references additional to those sourced from the panTHERIA life history database [35]. Click here for file

Additional file 4 **Rate Variation Test outputs**. PDF document containing outputs of tests of rate variation in all datasets used, comparing a free-rate versus fixed rate models across trees. Click here for file

Additional file 5 **Weighted Z Test calculations**. Excel spreadsheet containing values and calculations for Weighted Z test of multiple comparisons. Click here for file
