# Supplementary material for: Diversification and the rate of molecular evolution: no evidence of a link in mammals
Source: BMC Evol Biol. 2011 Oct 4;11:286. doi: 10.1186/1471-2148-11-286 (PMC3205075; doi:10.1186/1471-2148-11-286)
Supplement: Additional file 2 — Phylogenies. PDF document containing phylogenies used for all analyses described in the main text. [file 1471-2148-11-286-S2.PDF]

Additional File 2: Phylogenies

A2.1: Mitochondrial Pairs (Approximately Family Level)

A2.2: Mitochondrial Pairs (Deep)

A2.3: Mitochondrial Pairs (Shallow)

A2.4: Nuclear Pairs (Mammalia)

A2.5: Nuclear Pairs (Eutheria)

A2.6: Nuclear Pairs (Metatheria)

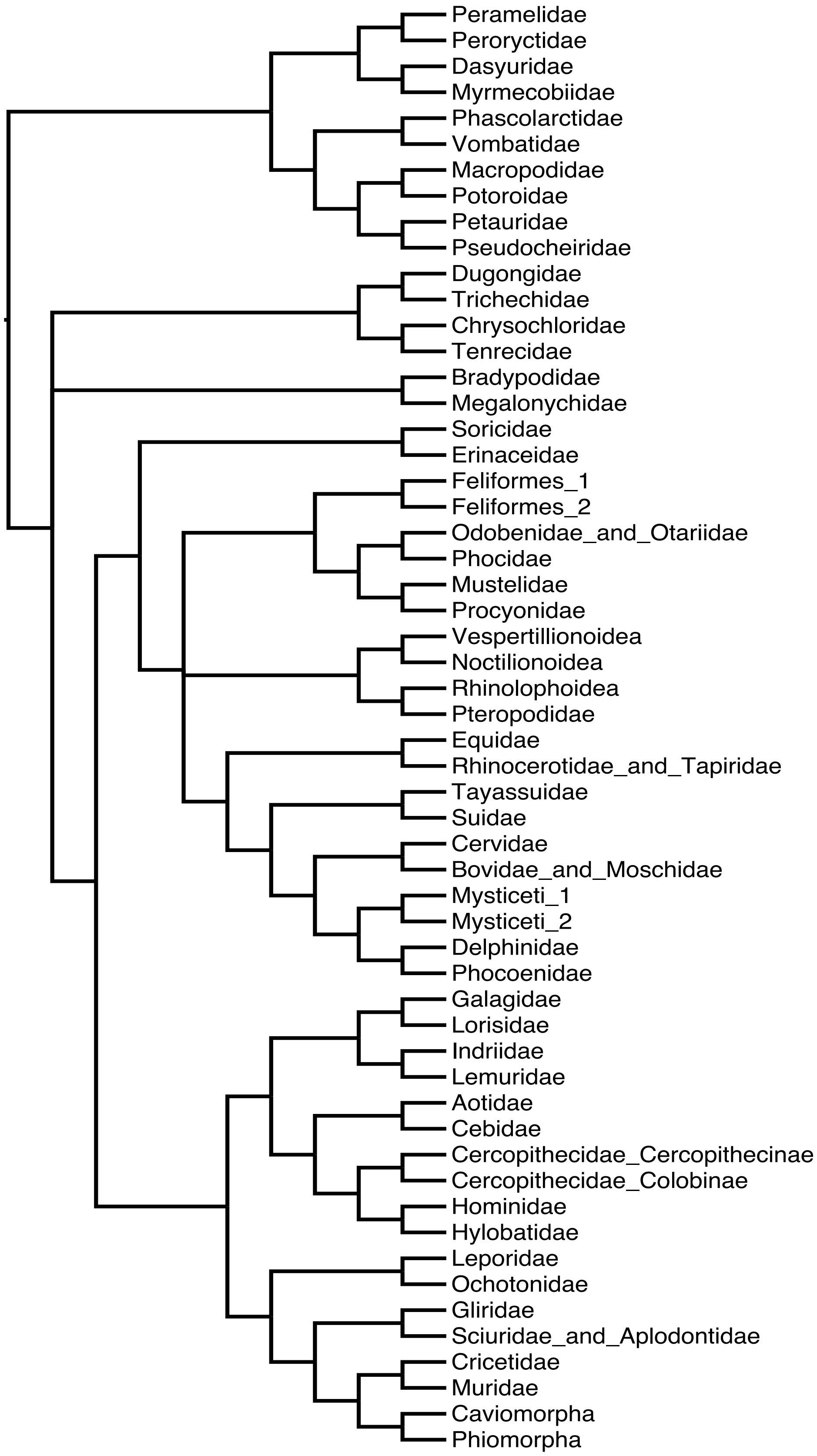

Figure A2.1: Phylogeny of family level (approximate) mitochondrial sister pairs (n=28). Total (**T**), synonymous (**dS**) and nonsynonymous (**dN**) substitution rates were estimated for each branch of the phylogeny. Ratios of **dN/dS** (**ω**) were estimated for each member of the sister pair. Feliformes 1: Felidae; Feliformes 2: Hyaenidae, Eupleridae, Viverridae and Herpestidae. Mysticeti 1: Balaenopteridae, Eschrichteridae, and Neobalaenidae. Mysticeti 2: Balaenidae

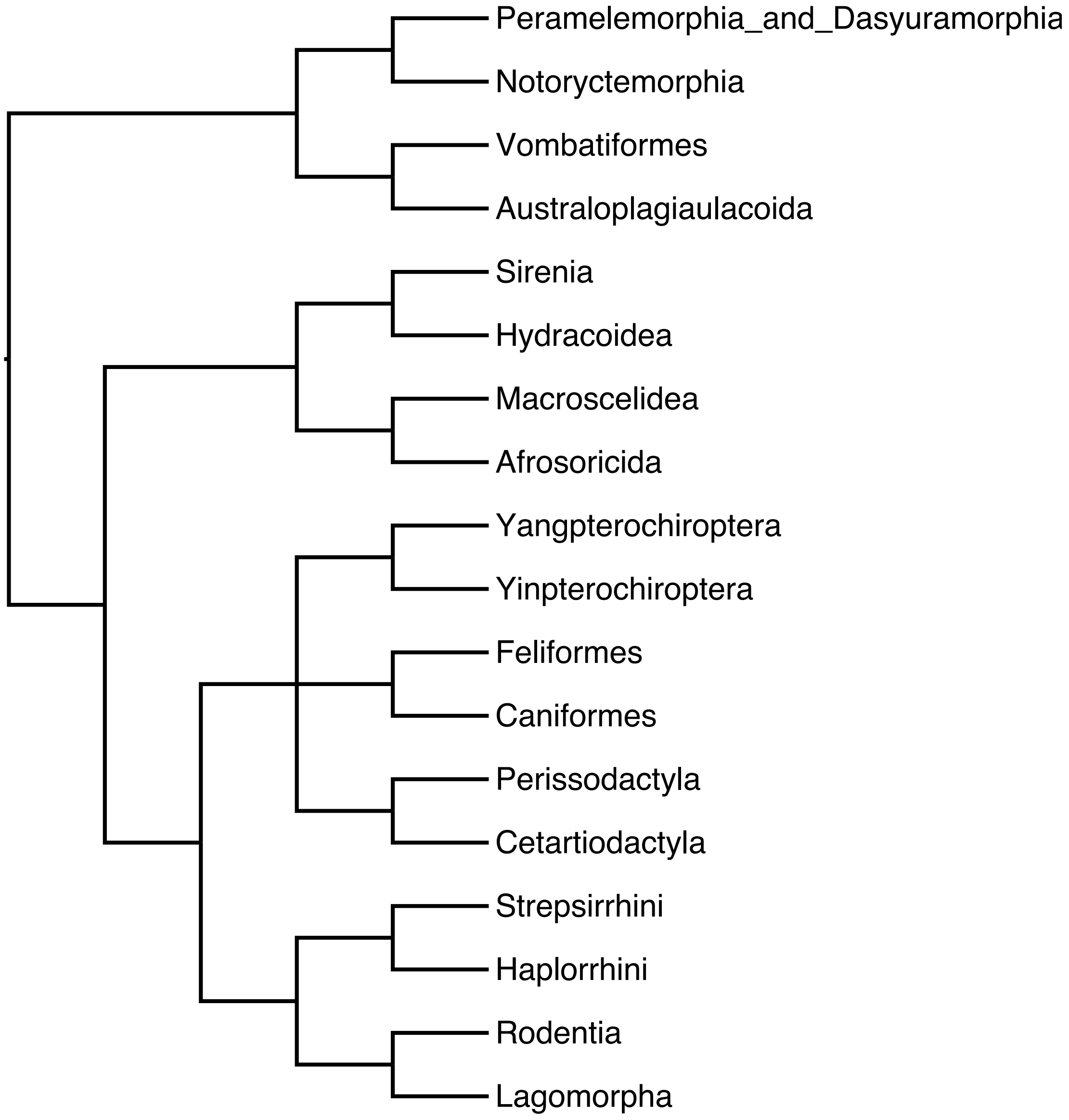

Figure A2.2: Phylogeny of deep mitochondrial sister pairs (n=9). Total (**T**), synonymous (**dS**) and non-synonymous (**dN**) substitution rates were estimated for each branch of the phylogeny. Ratios of **dN**/**dS** (**ω**) were estimated for each member of the sister pair.

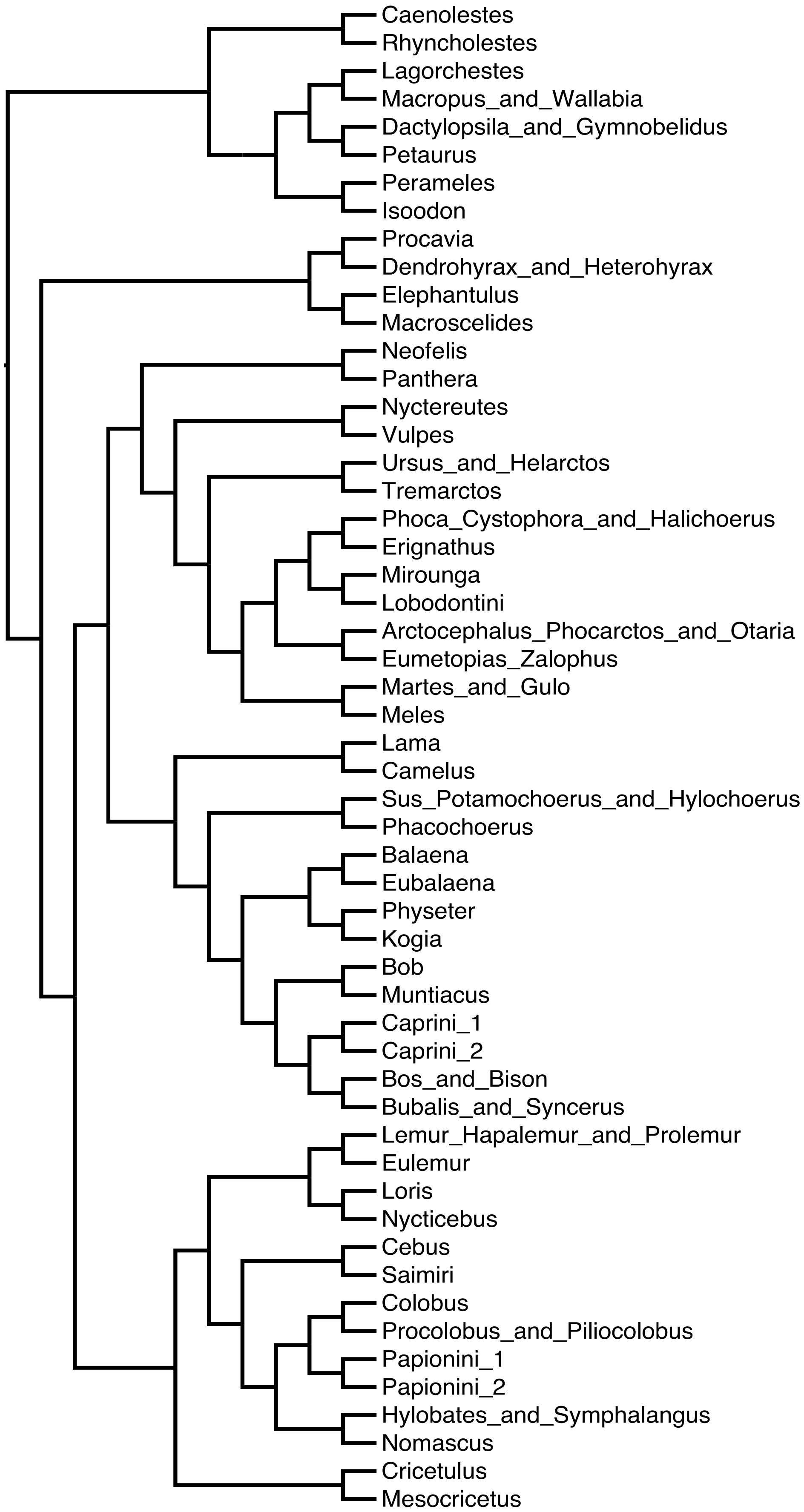

Figure A2.3: Phylogeny of shallow mitochondrial sister pairs (n=27). Total (**T**), synonymous (**dS**) and non-synonymous (**dN**) substitution rates were estimated for each branch of the phylogeny. Ratios of **dN**/**dS** (**ω**) were estimated for each member of the sister pair. Caprini_1: *Capra*, *Pseudois*, *Hemitragus*, *Ammotragus* and *Arabitragus*; Caprini_2: *Ovis*, *Rupicapra*, *Budorcas* and *Nilgiritragus*; Papioni_1: *Macaca*; Papionini_2: *Papio*, *Lophocebus*, *Theropithecus*, *Mandrillus* and *Cercocebus*.

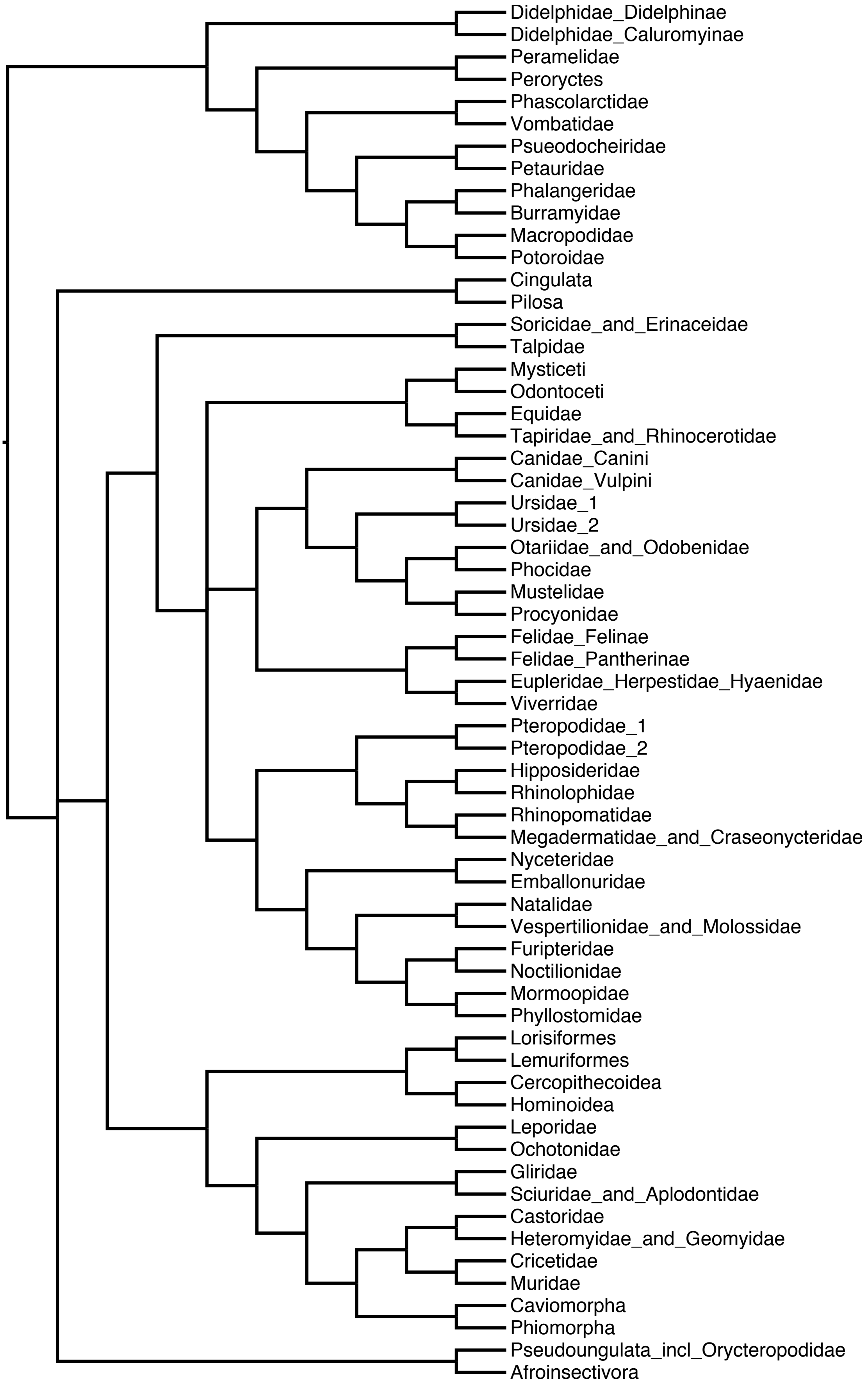

Figure A2.5: Phylogeny of nuclear mammalian sister pairs (n=31). Total (**T**), synonymous (**dS**) and non-synonymous (*dN*) substitution rates were estimated for each branch of the phylogeny. Ratios of *dN*/**dS** (**ω**) were estimated for each member of the sister pair. Ursidae_1: *Ailuropoda*; Ursidae_2: *Ursus*, *Helarctos* and *Tremarctos*; Feliformes_1: Eupleridae, Herpestidae and Hyaenidae; Feliformes_2: Viverridae; Pteropodidae_1: Nyctiminae and Cynopterinae; Pteropodidae_2: Harpiyonycterinae, Macroglossinae, Pteropodinae, Rousettinae and Epomophorinae;

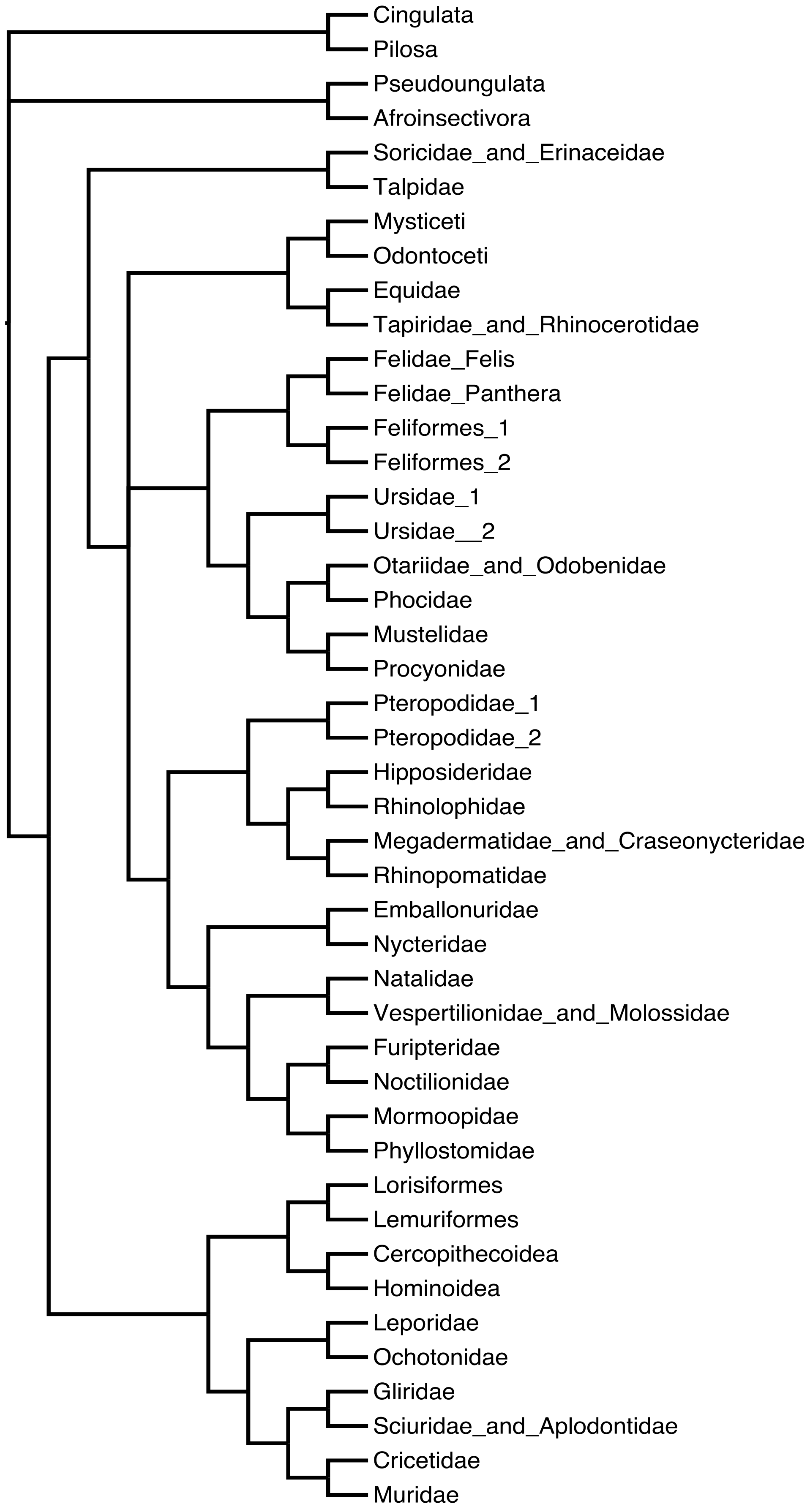

Figure A2.5: Phylogeny of nuclear eutherian sister pairs (n=22). Total (**T**), synonymous (**dS**) and non-synonymous (**dN**) substitution rates were estimated for each branch of the phylogeny. Ratios of **dN**/**dS** (**ω**) were estimated for each member of the sister pair. Ursidae_1: *Ailuropoda*; Ursidae_2: *Ursus*, *Helarctos* and *Tremarctos*; Feliformes_1: Eupleridae, Herpestidae and Hyaenidae; Feliformes_2: Viverridae; Pteropodidae_1: Nyctiminae and Cynopterinae; Pteropodidae_2: Harpiyonycterinae, Macroglossinae, Pteropodinae, Rousettinae and Epomophorinae;

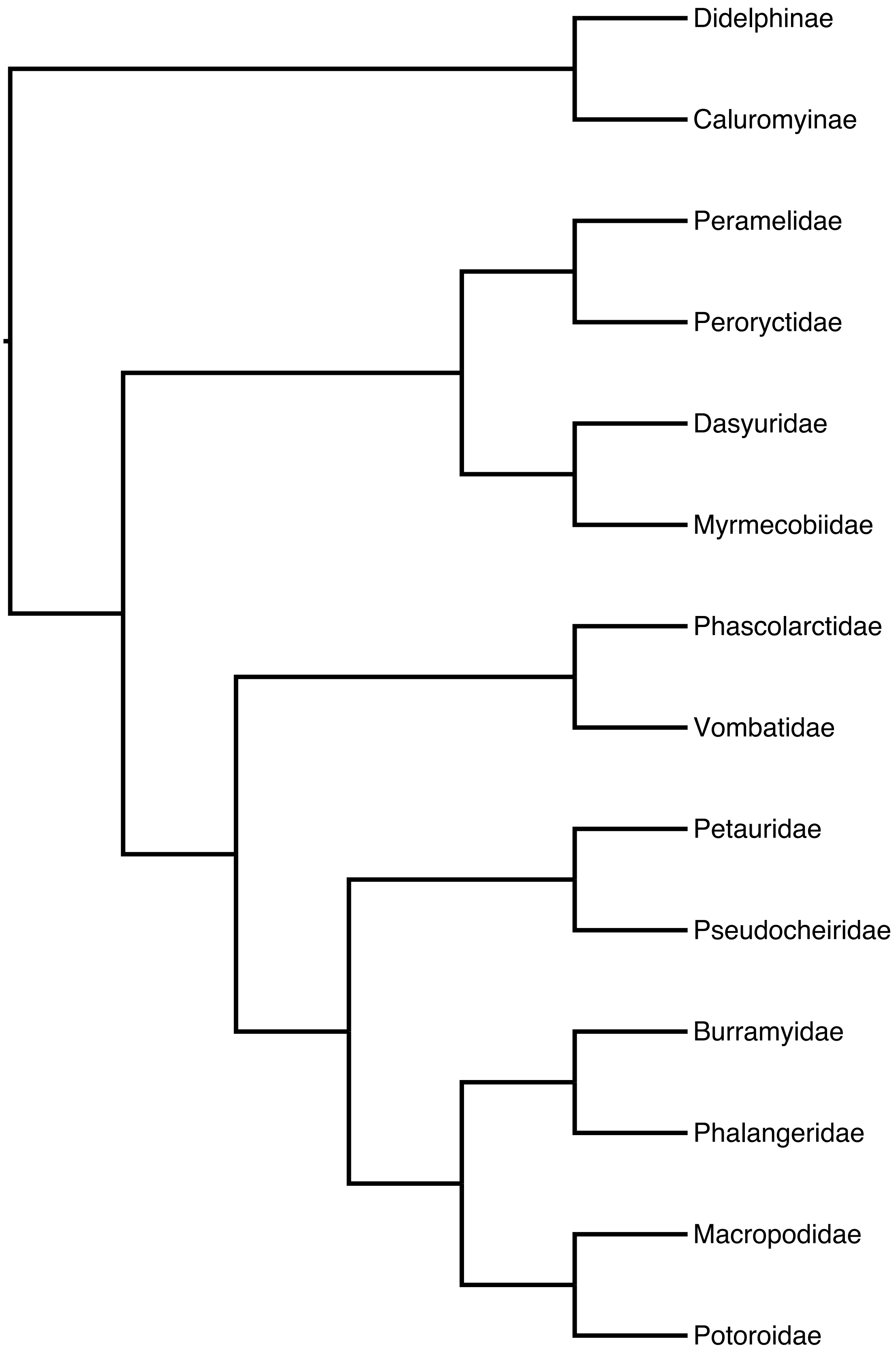

Figure A2.6: Phylogeny of nuclear metatherian sister pairs (n=7). Total (**T**), synonymous (**dS**) and non-synonymous (**dN**) substitution rates were estimated for each branch of the phylogeny. Ratios of **dN**/**dS** (**ω**) were estimated for each member of the sister pair.
