# Supplementary material for: Diversification and the rate of molecular evolution: no evidence of a link in mammals
Source: BMC Evol Biol. 2011 Oct 4;11:286. doi: 10.1186/1471-2148-11-286 (PMC3205075; doi:10.1186/1471-2148-11-286)
Supplement: Additional file 4 — Rate Variation Test outputs. PDF document containing outputs of tests of rate variation in all datasets used, comparing a free-rate versus fixed rate models across trees. [file 1471-2148-11-286-S4.PDF]

Additional File 4: Tests for Rate Variation

Alignment	Model	AIC	ΔAIC
Mitochondrial Family (Approx)	Free Rates	610735	-787
	Equal Rates	611522	
Mitochondrial Deep	Free Rates	232368	-360
	Equal Rates	232728	
Mitochondrial Shallow	Free Rates	550483	1130
	Equal Rates	549353	
Nuclear Mammalia	Free Rates	100645	-194
	Equal Rates	100839	
Nuclear Eutheria	Free Rates	101602	-359
	Equal Rates	101961	
Nuclear Metatheria	Free Rates	389606	787
	Equal Rates	388819	

Free Rates Model: where a separate substitution rate was estimated for each branch; Equal Rates Model: Terminal branches within each pair had equal substitution rates. AIC: Akaike Information Criterion Score calculated in *HyPhy* 2.0 for each model. ΔAIC: Difference in AIC scores (Free Rates - Equal Rates). Negative ΔAIC scores >10 indicate significantly better fit of model to free rates model. Positive ΔAIC scores >10 indicate significantly better fit of model to equal rates model
