# Supplementary material for: Diversification and the rate of molecular evolution: no evidence of a link in mammals
Source: BMC Evol Biol. 2011 Oct 4;11:286. doi: 10.1186/1471-2148-11-286 (PMC3205075; doi:10.1186/1471-2148-11-286)
Supplement: Additional file 5 — Weighted Z Test calculations. Excel spreadsheet containing values and calculations for Weighted Z test of multiple comparisons. [file 1471-2148-11-286-S5.PDF]

Additional File 5: Weighted Z Test Calculations

Hypothesis	Co-efficient	W (d.f.)	W^2	P Value	Left One Tailed P	Z Score
**Clade Size vs *T***						
Mitochondrial Medium	-0.6133	27	729	0.560	0.720	0.5828
Mitochondrial Deep	2.8130	8	64	0.358	0.179	-0.9192
Mitochondrial Shallow	-0.5455	25	625	0.683	0.659	0.4084
Nuclear Mammalia	-0.4149	23	529	0.645	0.678	0.4607
Nuclear Eutheria	1.8792	20	400	0.045	0.023	-2.0019
Nuclear Metatheria	-2.6421	6	36	0.401	0.800	0.8398
					**Weighted Z**	**-0.1190**
					**Combined P**	**0.4526**
**Clade Size vs *dN***						
Mitochondrial Medium	-1.1185	27	729	0.066	0.967	1.8384
Mitochondrial Deep	-0.6282	8	64	0.698	0.651	0.3880
Mitochondrial Shallow	0.2991	25	625	0.722	0.361	-0.3558
Welch et al. Mitochondrial*	-0.2485	42	1764	0.605	0.698	0.5172
Nuclear Mammalia	-0.5432	25	625	0.420	0.790	0.8064
Nuclear Eutheria	0.8154	18	324	0.312	0.156	-1.0110
Nuclear Metatheria	2.2613	6	36	0.304	0.152	-1.0279
					**Weighted Z**	**0.9506**
					**Combined P**	**0.8291**
**Clade Size vs *dS***						
Mitochondrial Medium*	-0.0865	16	256	0.236	0.882	1.1850
Mitochondrial Shallow*	-1.0500	23	529	0.107	0.947	1.6118
Welch et al. Mitochondrial*	-1.4968	26	676	0.096	0.952	1.6646
Nuclear Mammalia	0.0987	26	676	0.765	0.383	-0.2989
Nuclear Eutheria	1.2337	18	324	0.151	0.076	-1.4360
Nuclear Metatheria	-1.6842	6	36	0.220	0.890	1.2265
					**Weighted Z**	**1.4619**
					**Combined P**	**0.9281**
**Clade Size vs *ω***						
Mitochondrial Medium*	-0.0077	16	256	0.054	0.973	1.9268
Mitochondrial Shallow*	-0.0035	23	529	0.880	0.560	0.1510
Welch et al. Mitochondrial*	0.4371	27	729	0.423	0.212	-0.8012
Nuclear Mammalia	-1.2561	26	676	0.097	0.951	1.6586
Nuclear Eutheria	-1.6541	16	256	0.125	0.938	1.5341
Nuclear Metatheria	1.5536	6	36	0.130	0.065	-1.5141
					**Weighted Z**	**0.8970**
					**Combined P**	**0.8151**
**Clade Size vs Body Size**						
Mitochondrial Medium	0.1398	27	729	0.545	0.728	0.6068
Mitochondrial Deep	-0.4004	8	64	0.472	0.236	-0.7192
Mitochondrial Shallow	0.1566	24	576	0.416	0.792	0.8134
Welch et al. Mitochondrial	0.0783	42	1764	0.600	0.700	0.5244
Nuclear Mammalia	0.0414	30	900	0.793	0.604	0.2637
Nuclear Eutheria	-0.1453	21	441	0.412	0.206	-0.8204
Nuclear Metatheria	0.7831	6	36	0.140	0.930	1.4758
					**Weighted Z**	**0.7700**
					**Combined P**	**0.7794**
**Body Size vs *dN***						
Mitochondrial Family	-0.0073	27	729	0.921	0.461	-0.0990
Mitochondrial Deep	-0.0117	8	64	0.927	0.464	-0.0920
Mitochondrial Shallow	0.0006	24	576	0.990	0.505	0.0130
Welch et al Mitochondrial	0.0545	42	1764	0.256	0.872	1.1359
Nuclear Mammalia	-0.1062	25	625	0.041	0.020	-2.0456
Nuclear Eutheria	-0.2412	18	324	0.007	0.003	-2.7214
Nuclear Metatheria	0.1231	6	36	0.181	0.910	1.3380
**Weighted Z (Mitochond.):**	**0.7970**		**Weighted Z (Nucl.)**	**-2.9344**	**Weighted Z (All)**	**-0.7400**
**Combined P (Mitochond.):**	**0.7873**		**Combined P (Nucl.)**	**0.0017**	**Combined P (All)**	**0.2297**
**Body Size vs *dS***						
Mitochondrial Medium*	-0.2046	16	256	0.846	0.423	-0.1942
Mitochondrial Shallow*	0.0280	23	529	0.683	0.659	0.4084
Welch et al. Mitochondrial*	-0.1263	25	625	0.031	0.016	-2.1571
Nuclear Mammalia	-0.1524	25	625	0.004	0.002	-2.8782
Nuclear Eutheria	-0.0777	18	324	0.040	0.020	-2.0589
Nuclear Metatheria	-0.0644	6	36	0.679	0.340	-0.4138
					**Weighted Z**	**-3.2518**
					**Combined P**	**0.0006**
**Body Size vs *T***						
Mitochondrial Medium	0.0017	27	729	0.968	0.516	0.0401
Mitochondrial Deep	-0.0339	8	64	0.602	0.301	-0.5215
Mitochondrial Shallow	-0.0444	24	576	0.123	0.062	-1.5423
Nuclear Mammalia	-0.1237	22	484	0.002	0.001	-3.0902
Nuclear Eutheria	-0.0925	20	400	0.021	0.010	-2.3116
Nuclear Metatheria	-0.0094	6	36	0.895	0.448	-0.1320
					**Weighted Z**	**-3.2421**
					**Combined P**	**0.0006**
**Body Size vs *ω***						
Mitochondrial Medium*	-0.2721	16	256	0.13	0.935	**1.5141**
Mitochondrial Shallow*	0.028	23	529	0.683	0.3415	**-0.4084**
Welch et al Mitochondrial*	0.0586	36	1296	0.269	0.1345	**-1.1054**
Nuclear Mammalia	0.0287	26	676	0.529	0.2645	**-0.6295**
Nuclear Eutheria	0.1272	15	225	0.134	0.067	**-1.4985**
Nuclear Metatheria	0.1876	6	36	0.335	0.1675	**-0.9641**
					**Weighted Z**	**-1.2667**
					**Combined P**	**0.1026**

Hypothesis: independent tests of the same hypothesis are combined for the purposes of each Weighted Z test; Co-efficient: value of regression co-efficient for tests of each hypothesis; W: weighting, measured by degrees of freedom (d.f.) for each test of the hypothesis. P value: statistical signifcance of each parametric regression, from a two tailed t-test; Left one tailed P; P value converted to a left-tailed value in a one tailed distribution. For positive molecular rate versus clade size regression co-efficients, this value is half of the P value. For negative molecular rate versus clade size regression co-efficients, this value is (1 - half of the P value). For negative molecular rate versus body size regression co-efficients, this value is half of the P Value. For positive molecular rate versus body size regression co-efficients, this value is (1 -half of the P value); Z score: standardised Z scores for each one left one tailed P value; Weighted Z: Weighted average Z scores for combined tests of the same hypothesis, weighted by a measure of each test's degrees of freedom (W^2); Combined P: P Values for Weighted Z scores for each combined hypothesis. * Indicates regressions performed on rate estimates produced using PAML
